# Lineage-specific Nrf2 signaling orchestrates distinct neuroprotective mechanisms in acute ischemic stroke

**DOI:** 10.1038/s41419-026-09047-8

**Published:** 2026-07-13

**Authors:** Xiaoxiao Li, Chenchen Xu, Wenxin Xia, Caiyu Yang, Xin Wang, Jianjian Dong, Yongsheng Han

**Affiliations:** 1 Anhui University of Chinese Medicine, Hefei, China; 2 Institute of Neurology , Anhui University of Chinese Medicine, Hefei, China; 3 Center for Xin’an Medicine and Modernization of Traditional Chinese Medicine of IHM, Anhui University of Chinese Medicine, Heifei, China; 4 Wannan Medical University, Wuhu, China

**Keywords:** Stroke, Cell death in the nervous system

## Abstract

Nuclear factor erythroid 2–related factor 2 (Nrf2), a key antioxidant transcription factor, shows neuroprotective potential in ischemic stroke (IS); however, its cell type-specific functions across different neural lineages remain partially understood. This study innovatively employs a comparative knockout paradigm, utilizing neural lineage knockout (Nrf2^flox/flox^; Nestin-Cre, targeting neural progenitor cells and their derived lineages) and astrocyte-biased knockout (Nrf2^flox/flox^; GFAP-Cre) mouse models, combined with an in vitro co-culture system, to elucidate the lineage-dependent and differential protective mechanisms of Nrf2 in acute IS (AIS). Results demonstrated that both knockout models exacerbated neurological deficits, increased cerebral infarct volumes, and reduced cerebral blood flow. However, a marked phenotypic divergence was observed. The Nestin-Cre model exhibited more severe neurological deterioration, associated with dysregulated iron metabolism, enhanced lipid peroxidation, and aggravated neuroinflammation, suggesting a predominant role for neuronal Nrf2 in counteracting ferroptosis and neuroinflammatory responses. In contrast, the GFAP-Cre model did not induce ferroptosis but promoted neurotoxic A1-type astrocyte polarization and enhanced inflammatory injury via NF-κB pathway activation. This finding underscores the unique function of astrocytic Nrf2 in modulating the neuroinflammatory microenvironment. These cell-type-specific effects were further validated in an in vitro oxygen-glucose deprivation/reoxygenation (OGD/R) model. Through this cross-lineage comparative analysis, our study systematically elucidates, for the first time, the distinct protective mechanisms of Nrf2 in neurons and astrocytes, thereby advancing understanding of its functional heterogeneity and providing a novel theoretical basis for developing cell-type-biased, Nrf2-targeted therapeutic strategies.

**Figure:**
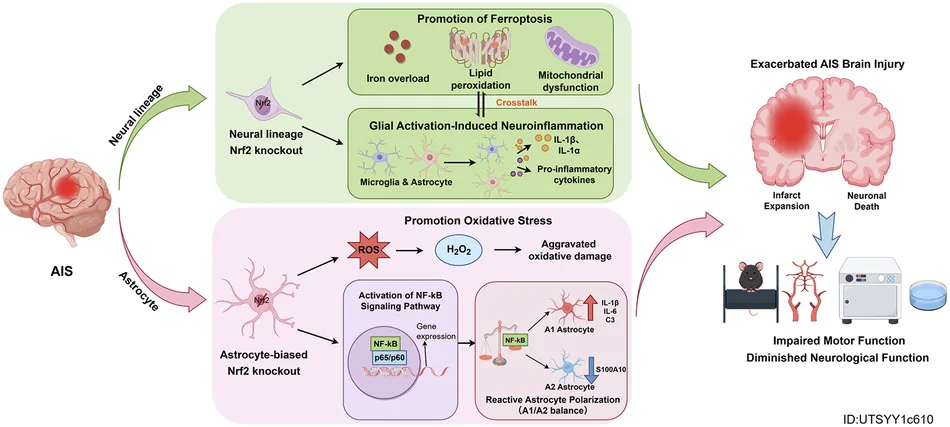

## Introduction

Stroke is the second leading cause of death worldwide and the third largest contributor to combined death and disability [1], representing a major component of the global disease burden [2]. Epidemiological data show that stroke-related deaths rose from 2.04 million in 1990 to 3.29 million in 2019 and are projected to reach 4.90 million by 2030 [3]. Acute ischemic stroke (AIS), accounting for approximately 87% of all stroke cases [4], is a medical emergency characterized by disrupted cerebral blood flow (CBF) and subsequent neuronal injury, often resulting in severe neurological deficits. Intravenous thrombolysis and mechanical thrombectomy are standard reperfusion therapies that significantly reduce AIS-related disability and mortality rates [5]. However, nearly half of patients who achieve vascular recanalization fail to regain functional independence, largely due to ischemia/reperfusion (I/R) injury [6]. Both ischemia and subsequent reperfusion trigger intense oxidative stress and inflammation, exacerbating irreversible neuronal damage [7].

Nuclear factor erythroid 2-related factor 2 (Nrf2), a key transcription factor, regulates antioxidant and anti-inflammatory responses and exerts neuroprotective effects in ischemic stroke (IS) by modulating ferroptosis-related pathways [8]. Studies show that Nrf2 activation mitigates oxidative stress, suppresses ferroptosis, and dampens neuroinflammation, thereby improving neurological recovery post-IS [9, 10]. Notably, Nrf2 activation exhibits context-dependent effects that vary with neural lineages and disease stage [11, 12]. Recent studies reveal spatiotemporal specificity in Nrf2 expression and function across different neural lineages during IS progression [13, 14]. Although substantial evidence indicates that Nrf2 exerts critical neuroprotective effects in neurons and astrocytes, most studies focus on single cell types or systemic Nrf2 modulation, with no comparative analyses across genetically defined lineages. Therefore, determining whether Nrf2 exerts distinct protective mechanisms in broad neural lineages versus astrocyte-biased populations is essential for understanding the cellular and molecular pathology of IS and for developing lineage-biased targeted therapeutic strategies.

This study utilizes Nestin-Cre and GFAP-Cre–mediated conditional knockout models, combined with a neuron–astrocyte co-culture system, to systematically compare the differential roles of Nrf2 within these genetically distinct lineages during acute ischemic brain injury. The study aims to uncover the underlying lineage-dependent molecular mechanisms, thereby providing a theoretical and experimental basis for advancing cell type-biased Nrf2-targeted therapies.

## Materials and methods

### Animals and grouping

Neural lineage (Nrf2^f/f^; Nestin-Cre) and astrocyte-biased (Nrf2^f/f^; GFAP-Cre) Nrf2 knockout mice were generated by crossing Nrf2^flox/flox^ (Nrf2^f/f^) homozygous mice (Shanghai Model Organisms Center) with Nestin-Cre or GFAP-Cre driver lines, respectively. It is important to note that the Nestin promoter is primarily active in neural precursor cells, Nestin-Cre–mediated recombination may affect neurons, astrocytes, and oligodendrocytes [15]. To assess the recombination efficiency, we performed immunofluorescence analysis. In Nrf2^f/f^; Nestin-Cre mice subjected to ischemic insult, Nrf2 expression was markedly reduced in neurons (NeuN⁺). However, a subset of astrocytes (GFAP⁺) retained Nrf2 expression (Fig. S1A). This observation may reflect inherent variability in recombination efficiency (chimerism) within this mouse line or could indicate that the pool of reactive astrocytes proliferating in response to ischemia originates from cells that escaped recombination, rather than being a model-specific artifact. Consequently, we objectively define the Nrf2^f/f^; Nestin-Cre model in this study as a neural lineage knockout. The phenotypic outcomes are interpreted as being predominantly driven by the loss of neuronal Nrf2, while acknowledging the potential for a contributory role from affected glial cells.

All male mice were 4–5 months old, weighed 25–30 g, and were housed under standard laboratory conditions at 24–26 °C with 50–60% relative humidity and a 12-h light/dark cycle, with food and water provided ad libitum. All procedures complied with the NIH Guide for the Care and Use of Laboratory Animals and were approved by the Animal Ethics Committee of Anhui University of Chinese Medicine (AHUCM-mouse-2023131). Mice were randomly assigned to the following groups: Nrf2^f/f^, Nrf2^f/f^ + MCAO/R (Nrf2^f/f^ + M/R), Nrf2^f/f^;Nestin-Cre (Nrf2^f/N^), Nrf2^f/N^ + MCAO/R (Nrf2^f/N^ + M/R), Nrf2^f/f^;GFAP-Cre (Nrf2^f/G^), Nrf2^f/G^ + MCAO/R (Nrf2^f/G^ + M/R), Nrf2^f/f^; Nestin-Cre+MCAO/R+Fer-1 (Nrf2^f/N^ + M/R+Fer-1), and Nrf2^f/f^; GFAP-Cre +MCAO/R+Fer-1 (Nrf2^f/G^ + M/R+Fer-1). The sample size was selected based on commonly used group sizes in MCAO/R studies and preliminary experimental data, which indicated a consistent trend in outcome measures [16, 17].

### Middle cerebral artery occlusion/reperfusion model establishment and drug intervention

The AIS model was established using the middle cerebral artery occlusion and reperfusion (MCAO/R) method [18]. Briefly, an infarction was induced in the left middle cerebral artery territory. Mice were anesthetized via intraperitoneal injection of 3% sodium pentobarbital (30 mg/kg), and body temperature was maintained at 37.0 ± 0.5°C using a heating pad throughout the procedure. After disinfecting the cervical area with povidone-iodine, a 1-cm midline incision was made. The underlying musculature was bluntly separated to expose the common, external, and internal carotid artery. The proximal common and external carotid arteries were ligated with silk sutures, and the internal carotid artery was temporarily occluded using an arterial clip. A small arteriotomy was created in the common carotid artery, and a standardized nylon filament (1220-A5, Beijing Cinontech Co., Ltd) was inserted into the internal carotid artery and advanced 10 ± 1 mm beyond the carotid bifurcation. After 60 min of occlusion, the filament was withdrawn to initiate reperfusion. The incision was closed in layers. Mice were then returned to their home cages. Neurological function was assessed after recovery using the modified Zea Longa scoring system [19], with scores of 1–3 indicating successful model establishment. Ferrostatin-1 (Fer-1, S7243, Selleck, USA) was administered intraperitoneally (10 mg/kg) at 24 h and 2 h before MCAO/R surgery [20]. Specimens were collected 24 h post-reperfusion according to the experimental protocol.

### Behavioral assessment

All mice underwent behavioral assessments 24 h after model induction, using a double-blind design employed to minimize bias.

#### Neurological deficit scoring

Neurological deficits were evaluated using the modified Longa scoring system: 0, no deficit; 1, mild flexion of the right forelimb; 2, rightward circling; 3, severe gait impairment with right-sided falling; and 4, loss of spontaneous locomotion or consciousness.

#### Rotarod test

Motor coordination and endurance were assessed using a Rotarod apparatus. Mice completed three trials per day (5 min each, separated by 30-min rest intervals) for 3 consecutive days. The first 2 days served as acclimatization training. During each trial, the rotation speed increased from 5 to 40 rpm. Latency to fall (maximum of 300 s) was recorded, and the mean of the three trials was used as the final measure.

### Laser speckle contrast imaging (LSCI) for cerebral blood flow (CBF)

CBF was monitored in real time using a laser speckle contrast imaging system (moorFLPI-2, Moor Instruments, UK). Mice were anesthetized with 4% isoflurane, secured in a stereotaxic frame, and the skull was surgically exposed. Baseline perfusion images were captured at 0.1 Hz using a 250-frame temporal filter (5 s per frame). Regional blood flow in the left middle cerebral artery territory was quantified in arbitrary perfusion units using a 12-color scale.

### Cells and grouping

The murine hippocampal neuronal cell line HT-22 and the astrocytic cell line C8-D1A were obtained in KeyGEN BioTECH (Wuhan, China). To mimic the in vivo microenvironment, a Transwell co-culture system was used. Briefly, HT-22 cells (2.5 × 10⁵ cells/well) were seeded in the lower compartment of a 6-well plate, and C8-D1A cells (1.5 × 10⁵ cells/well) were seeded in the upper inserts. The co-culture system was maintained in high-glucose DMEM (11995-065, Gibco) supplemented with 10% fetal bovine serum (10099-141 C, Gibco) and 1% penicillin/streptomycin (HY-K1006, MCE). Based on this setup, cells were assigned to the following groups: Con (Control), Con+Fer-1, OGD/R, OGD/R+Fer-1, OGD/R + NC shRNA (HT-22), OGD/R + NC shRNA (C8-D1A), OGD/R+*Nrf2* shRNA (HT-22), and OGD/R+*Nrf2* shRNA (C8-D1A).

### Oxygen-glucose deprivation/reoxygenation and drug treatment

The OGD/R procedure was performed as previously described [21]. Cells in 12-well or 6-well Transwell co-culture plates were subjected to OGD by replacing the medium with glucose-free DMEM (Gibco, 11966-025) and incubating in a hypoxic chamber (GOLD-SIM W200IR, SIM InternationalGroupCO., LTD, USA.) flushed with 95% N₂ and 5% CO₂ at 37°C for 2 h. Subsequently, the medium was replaced with standard serum-containing medium, and cells were returned to a normoxic incubator (37 °C, 5% CO₂) for 24 h of reoxygenation. Fer-1 (1 μmol/L) was added 4 h before OGD/R. Cells in the Con and Con+Fer-1 groups were maintained under standard culture conditions throughout.

### Lentiviral shRNA transduction for *Nrf2* knockdown in HT-22 or C8-D1A cells

To knock down *Nrf2* in HT-22 and C8-D1A cells, lentiviral vectors encoding Nrf2-specific short hairpin RNA (shRNA) or non-targeting control shRNA were produced by Hanbio Biotechnology Co., Ltd. (Shanghai, China). Based on preliminary optimization, HT-22 and C8-D1A cells were transduced at a multiplicity of infection of 30. Knockdown efficiency was confirmed via reverse transcription quantitative PCR (RT-qPCR) and Western blot analysis.

### 2,3,5-triphenyltetrazolium chloride (TTC) staining

For infarct quantification, mice were euthanized with 3% sodium pentobarbital and decapitated, and their brains were rapidly extracted. Coronal Section (2 mm) were incubated in 1% TTC (G1017, ServiceBio) at 37 °C for 30 min in the dark, then fixed, imaged, and analyzed using ImageJ with grayscale thresholding to calculate infarct volume.

### Fluoro-Jade B (FJB) staining

FJB staining was performed on paraffin-embedded brain sections. After deparaffinization and rehydration, a hydrophobic barrier was applied, and sections were incubated overnight at 4 °C with FJB working solution (1:400 in 50% glacial acetic acid). Nuclei were counterstained with DAPI (8 min), dehydrated, cleared with xylene, and mounted. FJB-positive neurons were visualized using a fluorescence microscope (Nikon Eclipse C1, Japan) and quantified using ImageJ.

### Hematoxylin and eosin (H&E) staining

Brain sections were stained with hematoxylin and eosin following standard deparaffinization, graded ethanol dehydration, xylene clearing, and mounting with neutral resin. Images were captured using bright-field microscopy (Nikon Eclipse E100, Nikon).

### Transmission electron microscopy (TEM)

Mitochondrial ultrastructure in cortical neurons was examined by transmission electron microscopy (TEM; HT7800/HT7700, Hitachi, Japan). Tissue blocks (1 mm³) were fixed in 1% osmium tetroxide, dehydrated, embedded in epoxy resin, polymerized, and sectioned into 70-nm ultrathin slices. Sections were stained with uranyl acetate and lead citrate before imaging.

### CCK-8 assay

HT-22 and C8-D1A cells were seeded in Transwell compartments and pre-cultured for 24 h. Following treatment, CCK-8 solution (CT0001-B, SparkJade) equivalent to 10% of the medium volume was added, and cells were incubated for 1–4 h. After incubation, 100 μL of medium from each well was transferred to a 96-well plate, and optical density at 450 nm was measured using a microplate reader.

### Reactive oxygen species detection

Intracellular ROS were detected using the fluorescent probe DCFH-DA. After treatments, cells were incubated with DCFH-DA working solution in the dark for 30 min, then washed three times with phosphate-buffered saline. Fluorescence was observed, and images were acquired using a fluorescence microscope.

### Reverse transcription quantitative PCR (RT-qPCR)

Total RNA was extracted using the Tissue/Cell RNA Kit (AC0202-B, SparkJade) and reverse-transcribed into cDNA with the SPARKscript II Kit (AG0304-B, SparkJade). Gene amplification was performed using the 2×SYBR Green qPCR Mix (AH0104-B, SparkJade) on an ABI 7500 real-time PCR system (Applied Biosystems). Reactions followed the manufacturer’s protocol: initial denaturation at 94 °C for 2–3 min, followed by 40 cycles of 94 °C (10–20 s), 55–60°C (10–20 s), and 72°C (20–30 s). Relative mRNA expression levels were normalized to *β-actin* using the 2^−ΔΔCt^ method. Table 1 lists the primer sequences.

**Table 1:** Primer sequences.

Gene	Forward (5′-3′)	Reverse (5′-3′)
β-actin	GGCTGTATTCCCCTCCATCG	CCAGTTGGTAACAATGCCATGT
Nrf2	TCTTGGAGTAAGTCGAGAAGTGT	GTTGAAACTGAGCGAAAAAGGC
GPX4	GATGGAGCCCATTCCTGAACC	CCCTGTACTTATCCAGGCAGA
SLC7A11	GGCACCGTCATCGGATCAG	CTCCACAGGCAGACCAGAAAA
TfR1	GTTTCTGCCAGCCCCTTATTAT	GCAAGGAAAGGATATGCAGCA
ACSL4	CTCACCATTATATTGCTGCCTGT	TCTCTTTGCCATAGCGTTTTTCT
C3	CCAGCTCCCCATTAGCTCTG	GCACTTGCCTCTTTAGGAAGTC
GBP2	CTGCACTATGTGACGGAGCTA	GAGTCCACACAAAGGTTGGAAA
Serping1	TAGAGCCTTCTCAGATCCCGA	ACTCGTTGGCTACTTTACCCA
S100A10	TGGAAACCATGATGCTTACGTT	GAAGCCCACTTTGCCATCTC
CLCF1	GACTCGTGGGGGATGTTAGC	CTAAGCTGCGGAGTTGATGCT
PTGS2	TTCAACACACTCTATCACTGGC	AGAAGCGTTTGCGGTACTCAT
IL-1α	CGAAGACTACAGTTCTGCCATT	GACGTTTCAGAGGTTCTCAGAG
IL-1β	GCAACTGTTCCTGAACTCAACT	ATCTTTTGGGGTCCGTCAACT
IL-6	TAGTCCTTCCTACCCCAATTTCC	TTGGTCCTTAGCCACTCCTTC

### Western blot analysis

Ischemic cerebral tissue was homogenized in RIPA buffer (Cat# R0020, Solarbio) supplemented with PMSF (Cat# FJP0100, Solarbio) to extract total protein. Protein concentrations were measured using a BCA assay. Equal amounts (30 μg) were separated by SDS-PAGE and transferred onto PVDF membranes. Membranes were blocked with 5% non-fat milk and incubated overnight at 4 °C with primary antibodies against Nrf2 (Cat# 380773, 1:1000, Zen-Bio), GPX4 (Cat# 381958, 1:1000, Zen-Bio), ACSL4 (Cat# A20414, 1:1000, ABclonal), SLC7A11 (Cat# 26864-1-AP, 1:1000, Proteintech), TfR1 (Cat# A5865, 1:1000, ABclonal), HO-1 (Cat# A28140PM, 1:1000, ABclonal), NQO1 (Cat# ab34173, 1:1000, Abcam), HIF-1α (Cat# A11945, 1:1000, Zen-Bio), 4-HNE (Cat# AB46545, 1:1000, Abcam), C3 (Cat# A26625PM, 1:2000, ABclonal), S100A10 (Cat# 11250-1-AP, 1:1000, Proteintech), β-actin (Cat# GB15003, 1:5000, ServiceBio), and β-tubulin (Cat# GB152667-100, 1:5000, ServiceBio). Following TBST washes, membranes were incubated with horseradish peroxidase-conjugated secondary antibody (Cat# AC2501246022, 1:10,000, ServiceBio) at room temperature for 1.5 h. Protein bands were visualized using ECL substrate (Cat# G2161, ServiceBio) and imaged with a Tanon Chemiluminescence Imaging System (Tanon, China). Band intensities were quantified using ImageJ software.

### Immunofluorescence staining

Paraffin-embedded brain tissue sections were deparaffinized, rehydrated, and subjected to antigen retrieval using citrate buffer (pH 6.0) at 95 °C for 8 min. Following blocking with 3% BSA for 30 min at room temperature, sections were incubated overnight at 4 °C with primary antibodies against Nrf2 (1:1000,), ACSL4 (1:1000), GPX4 (1:1000), 4-HNE (1:1000), GFAP (GB11096, 1:1000, ServiceBio), Iba1 (GB113502, 1:1000, ServiceBio), NeuN (GB11138, 1:1000, ServiceBio), C3 (1:2000), S100A10 (1:1000), and NF-κB (GB11997, 1:2000, ServiceBio). After three washes with PBS, sections were incubated with species-specific secondary antibodies for 50 min at room temperature in the dark, followed by DAPI staining for nuclear visualization. Autofluorescence was quenched using a dedicated reagent. Finally, sections were sealed with an anti-fade mounting medium and imaged using a fluorescence microscope. Quantification was performed using ImageJ software.

### Measurement of superoxide dismutase (SOD), malondialdehyde (MDA), reduced glutathione (GSH), oxidized glutathione (GSSG), ferrous iron (Fe^2^^+^) levels, and lipid peroxidation

Levels of SOD (Cat# BC0175, Solarbio), MDA (Cat# BC0025, Solarbio), GSH (Cat# BC1175, Solarbio), GSSG (Cat# BC1185, Solarbio), Fe^2+^ (Cat# BC5415, Solarbio), and lipid peroxidation (C11-BODIPY 581/591 probe; Cat# HY-D1301, MCE) in ischemic cerebral tissues were measured according to the manufacturers’ protocols.

### Statistical analysis

Statistical analyses were conducted using GraphPad Prism 9 (GraphPad, La Jolla, CA, USA). Data are presented as mean ± standard deviation (SD). Normality and homogeneity of variances were assessed prior to analysis. For parametric data, unpaired two-tailed Student’s *t* tests or one-way ANOVA were applied for comparisons between two or multiple groups, respectively. When variances were unequal, Brown-Forsythe and Welch ANOVA tests were used. Nonparametric data were analyzed using the Kruskal–Wallis test. A *P*-value < 0.05 was considered statistically significant.

## Results

### Cell type-specific Nrf2 expression in acute ischemic stroke mouse models

Previous studies show that Nrf2, a redox-sensitive transcription factor, is upregulated under oxidative stress conditions and exerts a protective role [8]. To examine its cell-specific distribution in the AIS model, we performed immunofluorescence staining of mouse brain tissue. Nrf2 expression was predominantly neuronal, with lower levels in astrocytes, indicating cell type-specific differential expression under ischemic stress (Fig. S1B).

### Neural lineage Nrf2 knockout exacerbates brain injury in acute ischemic stroke mouse models

**Fig. 1:**
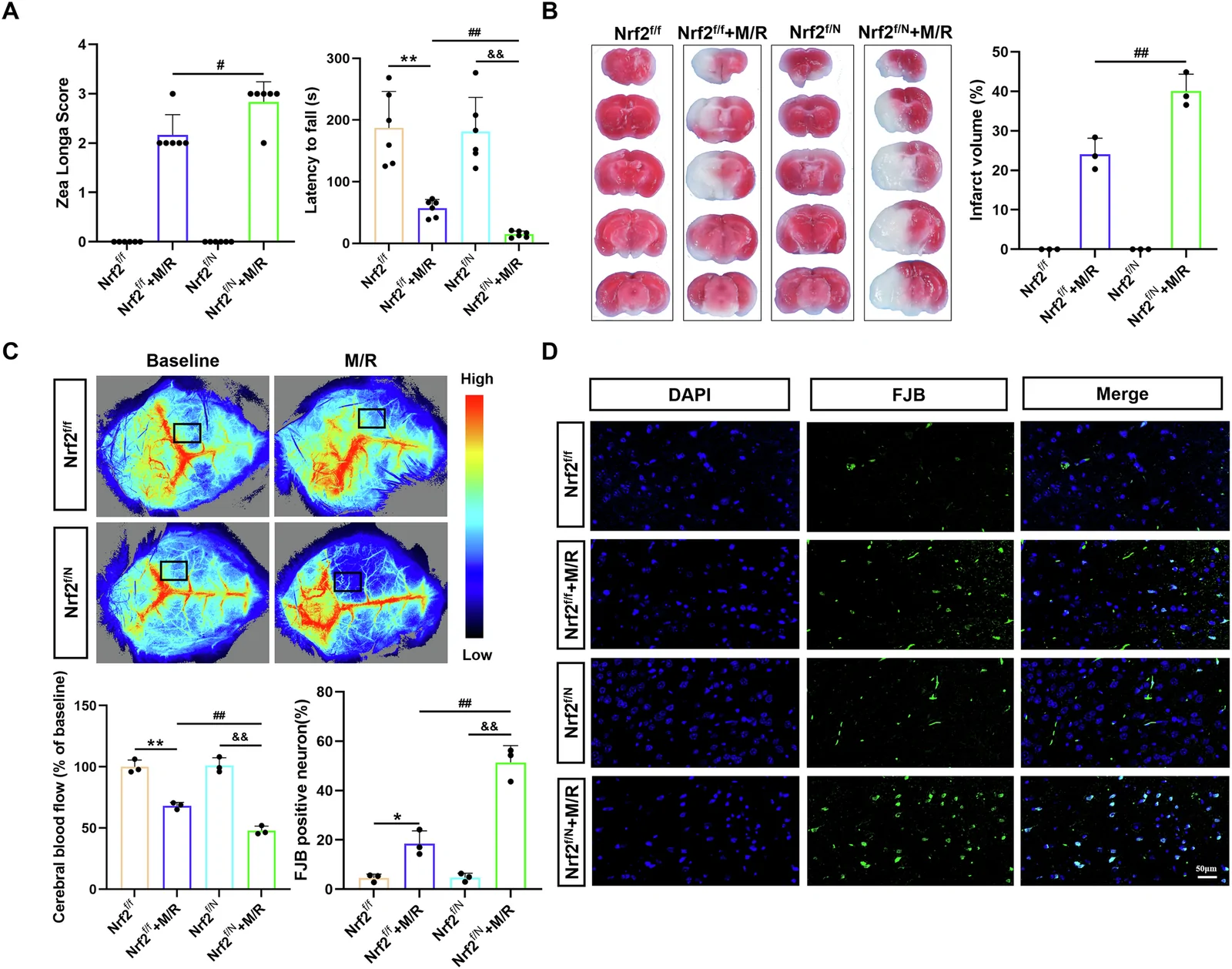
Neural lineage Nrf2 knockout exacerbates brain injury in AIS mouse models. **A** Neurological function assessed by Zea Longa scores and Rotarod tests (*n* = 6/group). **B** TTC staining showing cerebral infarction volume (*n* = 3/group). **C** Laser speckle imaging of cerebral blood flow (*n* = 3/group). **D** FJB-stained sections of the cerebral ischemic region in mice. Green indicates FJB-positive cells. Scale bar = 50 µm (*n* = 3/group). *P < 0.05, **P < 0.01 vs. Nrf2^f/f^ group; ^& &^*P* < 0.01 vs. Nrf2^f/N^ group; ^#^*P* < 0.05, ^##^*P* < 0.01 vs. Nrf2^f/f^ + M/R group.

Neurological function was assessed using Zea Longa scoring and Rotarod performance (Fig. 1A). Compared to Nrf2^f/f^ controls, the Nrf2^f/f^ + M/R group showed higher Zea Longa scores and shorter Rotarod latency, indicating neurological deficits. Neural lineage Nrf2 knockout further worsened AIS-related dysfunction in the Nrf2^f/N^ + M/R group. TTC staining (Fig. 1B) revealed a larger infarct volume in the Nrf2^f/N^ + M/R group than that in the Nrf2^f/f^ + M/R group. Laser speckle contrast imaging (Fig. 1C) showed reduced CBF in the ischemic hemisphere of the Nrf2^f/f^ + M/R group compared to controls, with a greater reduction in the Nrf2^f/N^ + M/R group. Fluoro-Jade B (FJB)staining (Fig. 1D) demonstrated an increased number of FJB-positive cells in the Nrf2^f/f^ + M/R group relative to Nrf2^f/f^ mice, and a further increase in the Nrf2^f/N^ + M/R group. Collectively, these results confirm that neural lineage Nrf2 deficiency aggravates neurological impairment, enlarges infarct volume, reduces cerebral perfusion, and exacerbates neuronal injury in the AIS mouse model. Based on the genetic profile of the model and co-staining results, this worsened phenotype is primarily driven by the absence of Nrf2 in neurons, while a potential contributory role from glial cells cannot be excluded.

### Astrocyte-biased Nrf2 knockout exacerbates cerebral injury in acute ischemic stroke mouse models

**Fig. 2:**
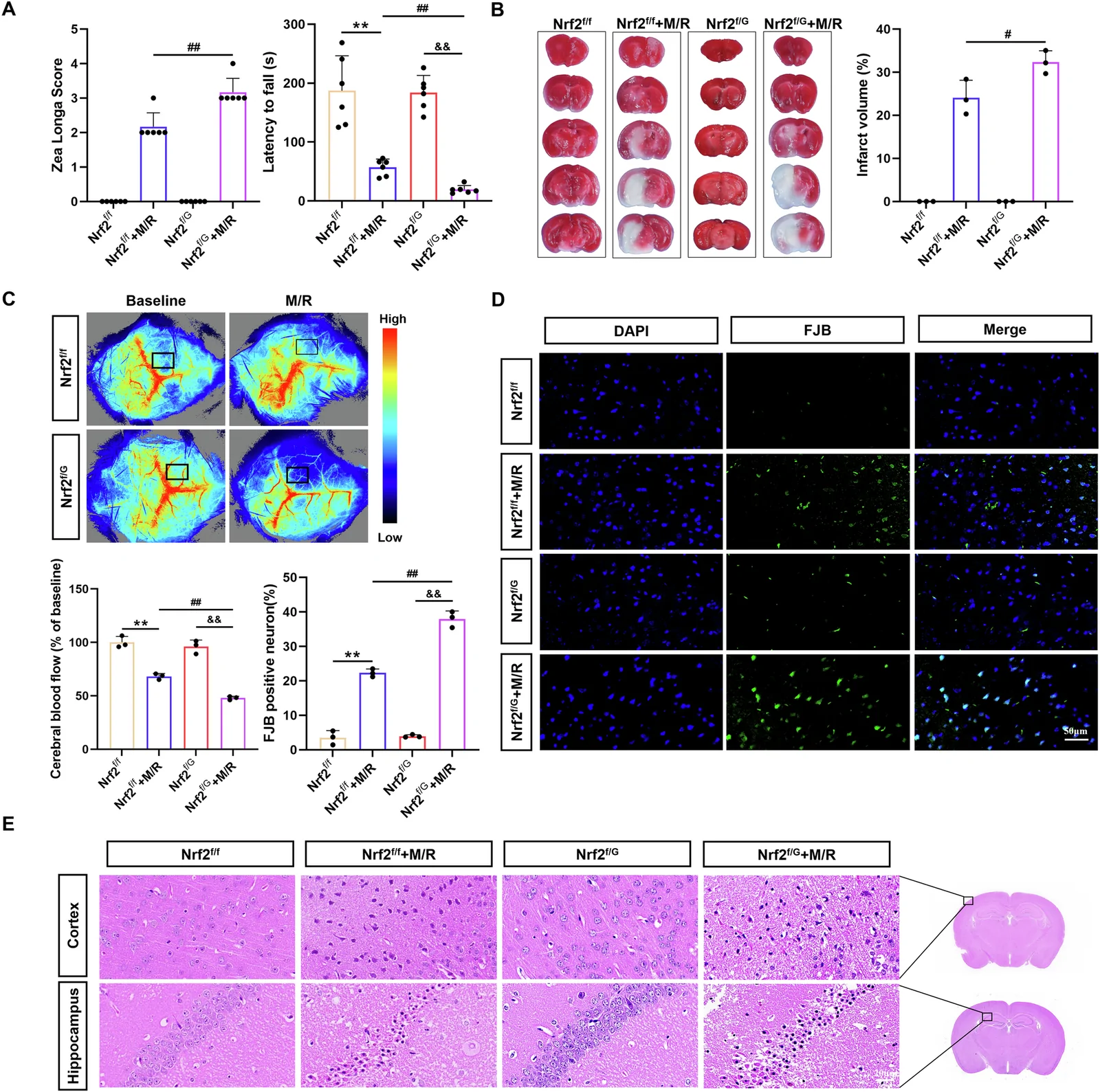
Astrocyte-biased Nrf2 knockout aggravates cerebral injury in AIS mouse models. **A** Neurological function assessed by the Zea Longa score and Rotarod test (*n* = 6/group). **B** Cerebral infarct volume measured by TTC staining (*n* = 3/group). **C** Cerebral blood flow monitored by laser speckle contrast imaging (*n* = 3/group). **D** FJB-stained sections of the cerebral ischemic region in mice. Green indicates FJB-positive cells. Scale bar = 50 µm (*n* = 3/group). **E** HE-stained sections of the injured cerebral cortex and hippocampal regions in each mouse group. Scale bar=20 µm(n = 3/group). **P < 0.01 vs. Nrf2^f/f^ group; ^& &^*P* < 0.01 vs. Nrf2^f/G^ group; ^#^*P* < 0.05, ^##^*P* < 0.01 vs. Nrf2^f/f^ + M/R group.

The effect of astrocyte-biased Nrf2 deletion on AIS outcomes was examined. Behavioral analyses (Fig. 2A) showed that the Nrf2^f/G^ + M/R group had higher Zea Longa scores and shorter Rotarod latency than the Nrf2^f/f^ + M/R group, indicating worsened neurological function. TTC staining (Fig. 2B) revealed a larger infarct volume in the Nrf2^f/G^ + M/R group. Laser speckle imaging (Fig. 2C) confirmed decreased cerebral perfusion in these mice relative to Nrf2^f/f^ + M/R controls. FJB staining (Fig. 2D) showed more FJB-positive cells in the Nrf2^f/G^ + M/R group, consistent with increased neuronal degeneration. In Nrf2^f/f^ and Nrf2^f/G^ mice, HE staining (Fig. 2E) revealed well-preserved neuronal morphology, with clear boundaries and compact cellular architecture in the cortex and hippocampus. In contrast, the Nrf2^f/f^ + M/R and Nrf2^f/G^ + M/R groups exhibited disrupted neuronal structure, disordered cell arrangement, and nuclear pyknosis. These findings indicate that astrocyte-biased Nrf2 knockout exacerbates neurological deficits, infarct size, perfusion deficits, neuronal injury, and histopathological damage in AIS mouse model. Given that GFAP-Cre-mediated recombination primarily targets the astrocytic lineage, these findings indicate that astrocytic Nrf2 serves a pivotal protective role in acute ischemic brain injury, and its loss contributes to and mediates the exacerbation of cerebral damage.

### Neural lineage and astrocyte-biased Nrf2 differentially regulate ferroptosis and oxidative damage in acute ischemic stroke mouse models

**Fig. 3:**
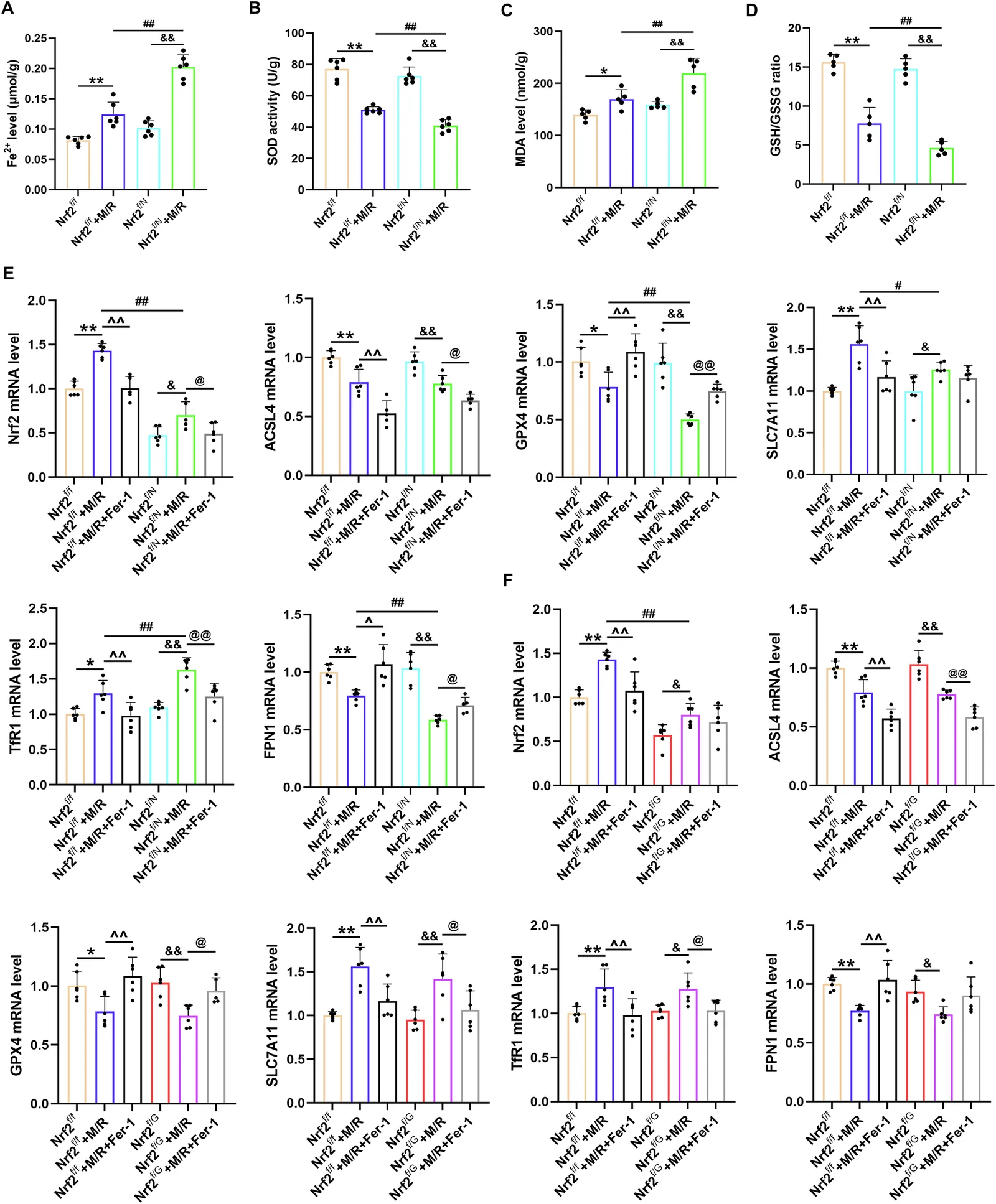
Neural lineage and astrocyte-biased Nrf2 differentially regulate ferroptosis and oxidative damage in AIS mouse models. **A** Fe²⁺ levels in the cerebral ischemic region (*n* = 6/group). **B** SOD activity (*n* = 6/group). **C** MDA levels (*n* = 5/group). **D** GSH/GSSG ratio (*n* = 5/group). **E** Effect of neural lineage Nrf2 knockout on mRNA expression of key ferroptosis factors (*Nrf2*, *ACSL4*, *GPX4*, *SLC7A11*, *TfR1*, *FPN1*; *n* = 5–6/group). **F** Effect of astrocyte-biased Nrf2 knockout on mRNA expression of key ferroptosis factors (*Nrf2*, *ACSL4*, *GPX4*, *SLC7A11*, *TfR1*, *FPN1*; *n* = 5–6/group). ^*^P < 0.05, ^**^P < 0.01 vs. Nrf2^f/f^ group; ^&^*P* < 0.05, ^& &^*P* < 0.01 vs. Nrf2^f/N^ or Nrf2^f/G^ group; ^#^*P* < 0.05, ^##^*P* < 0.01 vs. Nrf2^f/f^ + M/R group; ^@^*P* < 0.05, ^@@^*P* < 0.01 vs. Nrf2^f/N^ + M/R or Nrf2^f/G^ + M/R group; ^^^*P* < 0.05, ^^^^*P* < 0.01 vs. Nrf2^f/f^ + M/R+Fer-1 group.

Ferroptosis and oxidative stress-related markers in brain tissues were systematically analyzed across two genetically defined models to determine the role and regulatory mechanisms of lineage-dependent Nrf2 in AIS progression. Compared to the Nrf2^f/f^ group, the Nrf2^f/f^ + M/R group exhibited higher Fe²⁺ levels, lower superoxide dismutase (SOD) activity, increased malondialdehyde (MDA) levels, and a reduced glutathione/oxidized glutathione (GSH/GSSG) ratio. Lineage-specific Nrf2 knockout further exacerbated these alterations. Nrf2^f/N^ + M/R mice exhibited greater disruptions in all iron metabolism and oxidative stress indicators than Nrf2^f/f^ + M/R mice (Fig. 3A–D). In contrast, the Nrf2^f/G^ + M/R group exhibited only reduced SOD activity and increased MDA levels, with no significant changes in Fe²⁺ level or the GSH/GSSG ratio (Fig. S2). These findings indicate that neuronal Nrf2 primarily regulates ferroptosis in the AIS model, while astrocytic Nrf2 predominantly modulates the oxidative stress response.

**Fig. 4:**
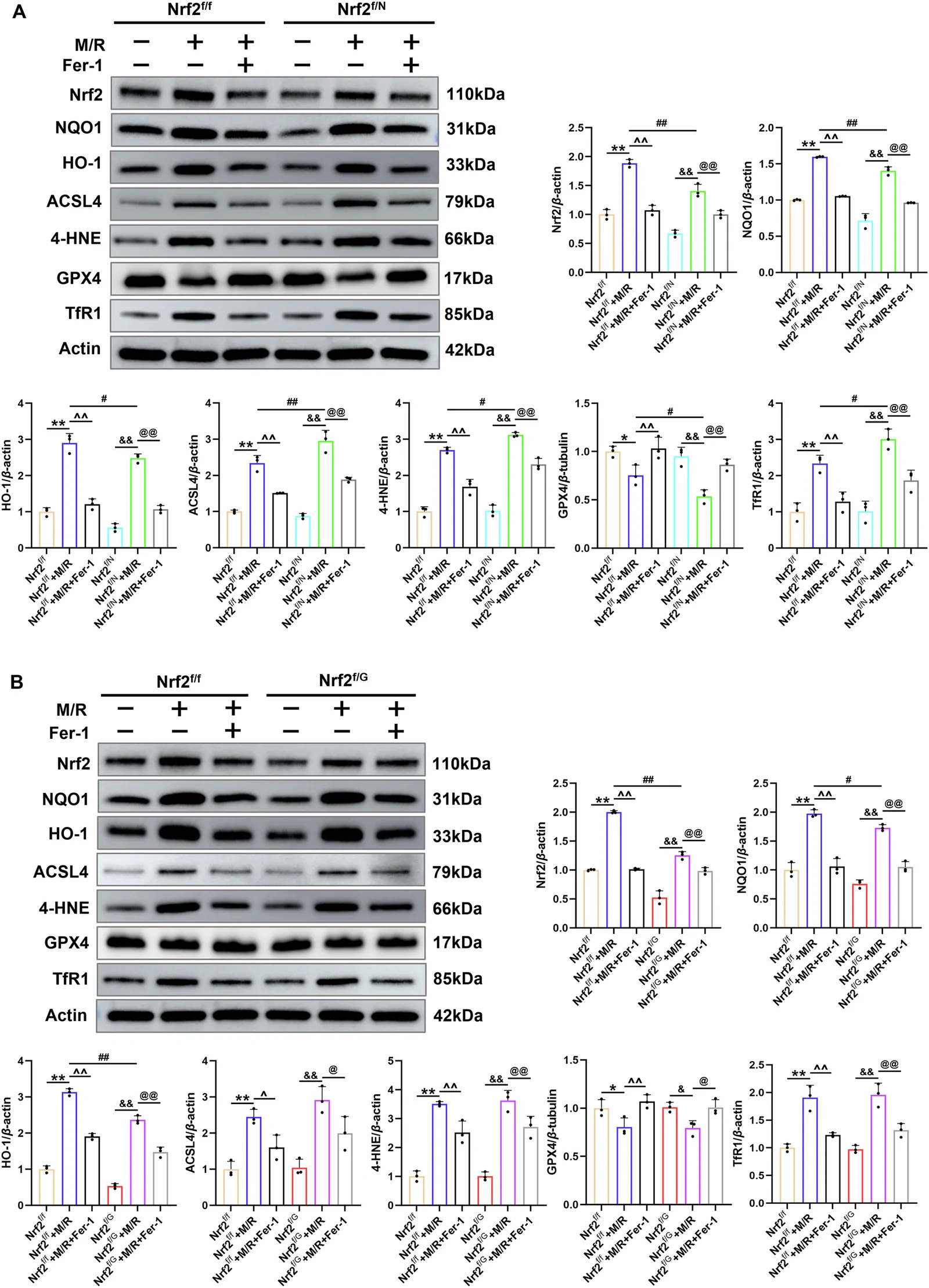
Neural lineage and astrocyte-biased Nrf2 exert differential modulation on the expression of ferroptosis-related proteins. **A** Western blot analysis of key ferroptosis proteins after neural lineage Nrf2 knockout (Nrf2, NQO1, HO-1, ACSL4, 4-HNE, GPX4, TfR1; *n* = 3/group). **B** Western blot analysis of key ferroptosis proteins after astrocyte-biased Nrf2 knockout (Nrf2, NQO1, HO-1, ACSL4, 4-HNE, GPX4, TfR1; *n* = 3/group). ^*^P < 0.05, ^**^P < 0.01 vs. Nrf2^f/f^ group; ^&^*P* < 0.05, ^& &^*P* < 0.01 vs. Nrf2^f/N^ or Nrf2^f/G^ group; ^#^*P* < 0.05, ^##^*P* < 0.01 vs. Nrf2^f/f^ + M/R group; ^@^*P* < 0.05, ^@@^*P* < 0.01 vs. Nrf2^f/N^ + M/R or Nrf2^f/G^ + M/R group; ^^^*P* < 0.05, ^^^^*P* < 0.01 vs. Nrf2^f/f^ + M/R+Fer-1 group.

The ferroptosis-specific inhibitor Fer-1 was used to test this hypothesis. RT-qPCR showed that Nrf2^f/N^ + M/R mice had higher *TfR1* mRNA and lower *FPN1* and *GPX4* mRNA expressions than Nrf2^f/f^ + M/R mice; Fer-1 treatment reversed these changes. In contrast, the Nrf2^f/G^ + M/R group showed no significant alterations in *TfR1*, *FPN1*, or *GPX4* expression (Fig. 3E, F). Western blot analysis confirmed that Nrf2^f/N^ + M/R mice had higher ACSL4, TfR1, and 4-HNE protein levels and lower GPX4 levels, which were reversed after Fer-1 treatment. Compared to the Nrf2^f/f^ + M/R group, the Nrf2^f/G^ + M/R group showed no significant differences in ACSL4, TfR1, GPX4, or 4-HNE protein levels (Fig. 4A, B). These findings align with the gene expression trends, indicating that neuronal Nrf2 primarily modulates ferroptosis in AIS models, whereas astrocytic Nrf2 regulates oxidative damage, reflecting cell type-specific functions.

M/R treatment upregulated *Nrf2*, and its downstream target genes *NQO1* and *HO-1*, while downregulating *ACSL4* mRNA, suggesting an endogenous protective response to AIS stress [22]. After Fer-1 intervention, Nrf2 expression decreased at the gene and protein levels, indicating that ferroptosis inhibition reduces the acute stress response. NQO1 and HO-1 expression increased post-M/R and decreased after Fer-1 treatment, consistent with Nrf2 expression and reflecting their roles as downstream targets. Furthermore, the discrepancy between decreased *ACSL4* mRNA and increased protein expression suggests post-transcriptional regulation, warranting further investigation.

**Fig. 5:**
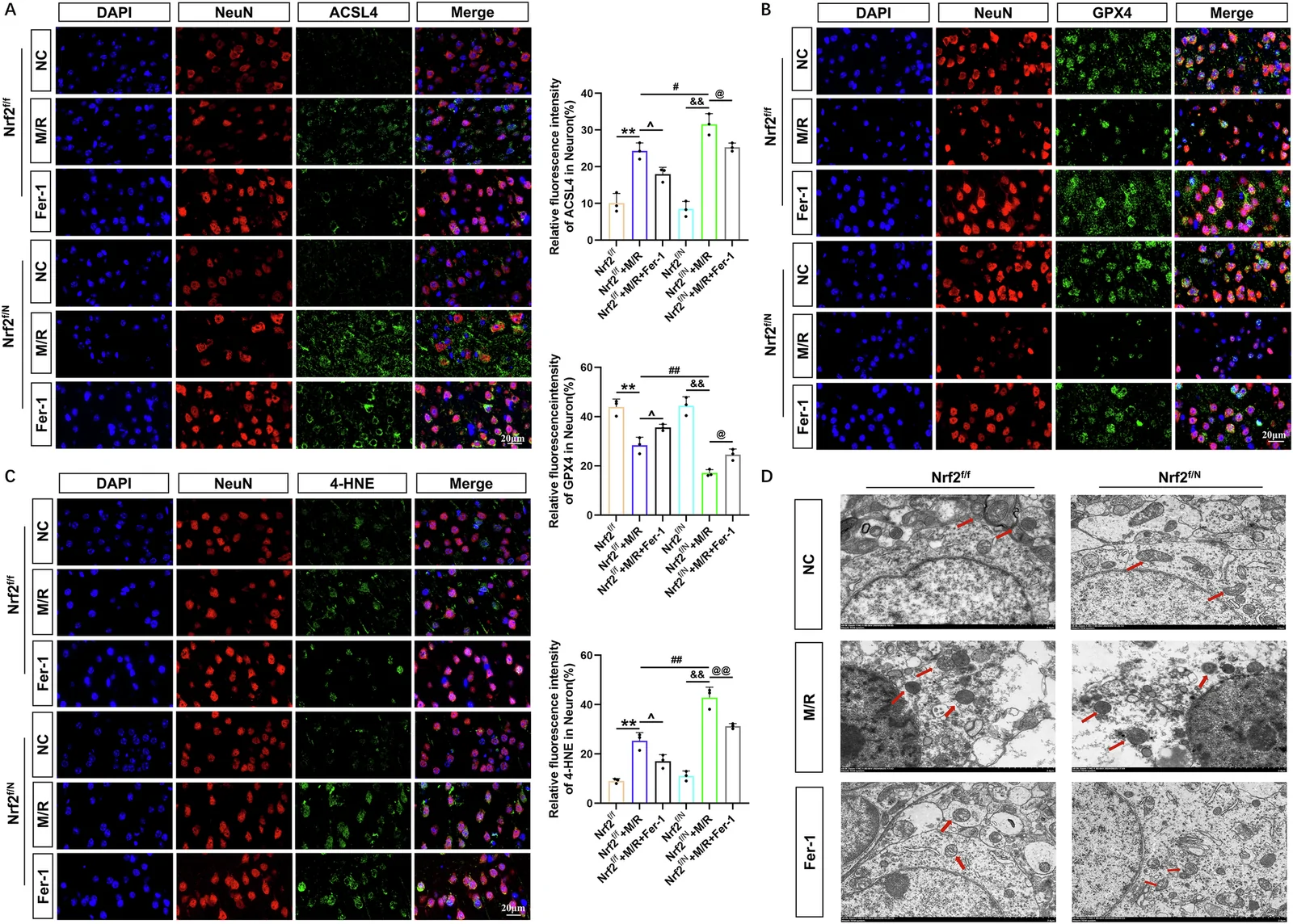
Neural lineage Nrf2 knockout promotes ferroptosis in AIS mouse models. Immunofluorescence staining of neurons (red) for ACSL4 (green, **A**), GPX4 (green, **B**), and 4-HNE (green, **C**) in the cerebral ischemic region. Scale bar = 20 µm (*n* = 3/group). **D** TEM images of neuronal mitochondria (red arrows). Scale bar = 5 µm/2 µm (n = 3/group). ^*^P < 0.05, ^**^P < 0.01 vs. Nrf2^f/f^ group; ^&^*P* < 0.05, ^& &^*P* < 0.01 vs. Nrf2^f/N^ or Nrf2^f/G^ group; ^#^*P* < 0.05, ^##^*P* < 0.01 vs. Nrf2^f/f^ + M/R group; ^@^*P* < 0.05, ^@@^*P* < 0.01 vs. Nrf2^f/N^ + M/R or Nrf2^f/G^ + M/R group; ^^^*P* < 0.05, ^^^^*P* < 0.01 vs. Nrf2^f/f^ + M/R+Fer-1 group.

To further confirm the regulatory role of neural lineage-specific Nrf2 in ferroptosis, the cell-specific expression of key ferroptosis factors ACSL4, GPX4, and 4-HNE in neurons was assessed via immunofluorescence staining, and ultrastructural changes in neuronal mitochondria were examined using transmission electron microscopy. Immunofluorescence results showed that neurons in the Nrf2^f/f^ + M/R group exhibited increased ACSL4 and 4-HNE fluorescence intensity and decreased GPX4 intensity compared to those in the Nrf2^f/f^ group. These changes were further exacerbated in the Nrf2^f/N^ + M/R group (Fig. 5A–C). Transmission electron microscopy revealed typical ferroptosis-like mitochondrial damage in the Nrf2^f/f^ + M/R group—including reduced mitochondrial volume, increased membrane density, outer membrane rupture, and loss or reduction of cristae—which was more severe in the Nrf2^f/N^ + M/R group. Fer-1 treatment effectively reversed these effects, decreasing ACSL4 and 4-HNE fluorescence, increasing GPX4 intensity in neurons, and significantly improving mitochondrial morphology (Fig. 5D).

Our findings from a cross-model comparison demonstrated a severe ferroptotic phenotype specifically upon neural lineage-selective Nrf2 deletion, in contrast to the lack of such a phenotype following astrocyte-biased Nrf2 ablation. This evidence supports the notion that neuronal Nrf2 deficiency is the dominant factor promoting ferroptosis in acute ischemic brain injury, while astrocytic Nrf2 loss is mainly linked to oxidative stress damage. The selective rescue of ferroptosis by Fer-1 exclusively in the Nestin-Cre model provides further validation for this comparative knockout strategy.

### Neural lineage Nrf2 knockout exacerbates hypoxia-inducible factor-1α upregulation and ferroptosis in acute ischemic stroke mouse models

**Fig. 6:**
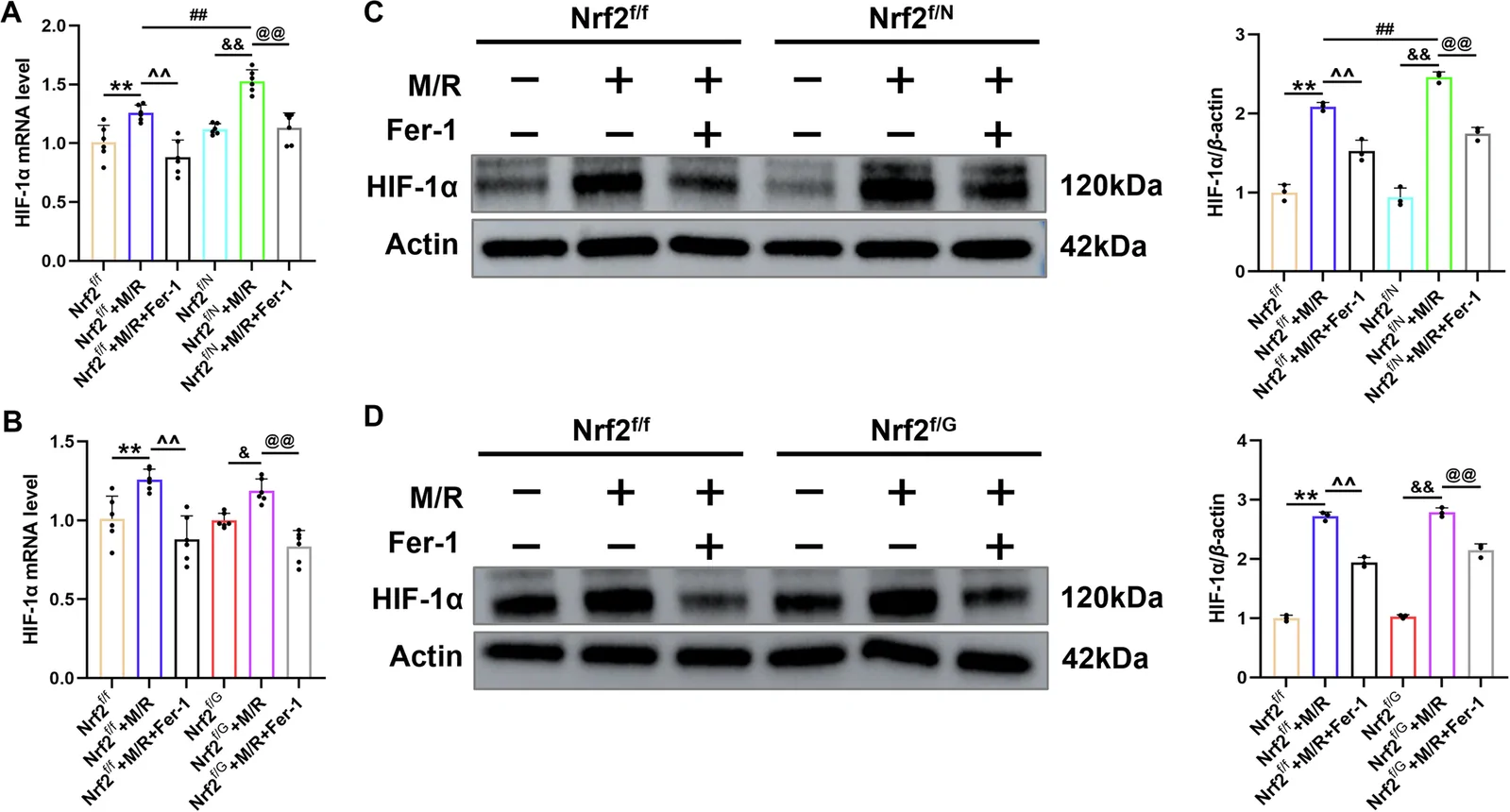
Neural lineage Nrf2 knockout upregulates HIF-1α and promotes ferroptosis in AIS mouse models. **A** Effect of neural lineage Nrf2 knockout on *HIF-1α* mRNA expression (*n* = 6/group). **B** Effect of astrocyte-biased Nrf2 knockout on *HIF-1α* mRNA expression (*n* = 6/group). **C** Western blot analysis of HIF-1α protein expression after neural lineage Nrf2 knockout (*n* = 3/group). **D** Western blot analysis of HIF-1α protein expression after astrocyte-biased Nrf2 knockout (*n* = 3/group). ^**^P < 0.01 vs. Nrf2^f/f^ group; ^&^*P* < 0.05, ^& &^*P* < 0.01 vs. Nrf2^f/N^ or Nrf2^f/G^ group; ^##^*P* < 0.01 vs. Nrf2^f/f^ + M/R group; ^@^*P* < 0.05, ^@@^*P* < 0.01 vs. Nrf2^f/N^ + M/R or Nrf2^f/G^ + M/R group; ^^^*P* < 0.05, ^^^^*P* < 0.01 vs. Nrf2^f/f^ + M/R+Fer-1 group.

Hypoxia-inducible factor-1α (HIF-1α) regulates oxygen homeostasis and increases rapidly after hypoxia/ischemia, influencing IS through multiple biological pathways [23]. Studies show that during IS, HIF-1α expression is cell type-specific and bidirectionally regulates ferroptosis [24–26]. We measured HIF-1α mRNA and protein levels to determine whether HIF-1α mediates the lineage-specific differences in Nrf2-regulated ferroptosis observed between the two genetic models. Compared to the Nrf2^f/f^ group, the Nrf2^f/f^ + M/R group showed significant upregulation of HIF-1α mRNA and protein, indicating that M/R treatment alone induces HIF-1α expression. This upregulation increased further in the Nrf2^f/N^ + M/R group, whereas no significant change was observed in the Nrf2^f/G^ + M/R group. Furthermore, Fer-1 treatment reduced HIF-1α expression, implicating its upregulation in the ferroptotic process (Fig. 6A−D). Collectively, these results indicate that HIF-1α is markedly activated in the AIS model and negatively regulated by neural lineage Nrf2, while astrocytic Nrf2 has no significant effect. Taken together with the Fer-1 intervention data, these findings suggest that the differential regulation of ferroptosis by neural versus astrocytic Nrf2 may involve distinct molecular mechanisms mediated by HIF-1α.

### Neural lineage Nrf2 knockout promotes glial cell activation and neuroinflammation in acute ischemic stroke mouse models

**Fig. 7:**
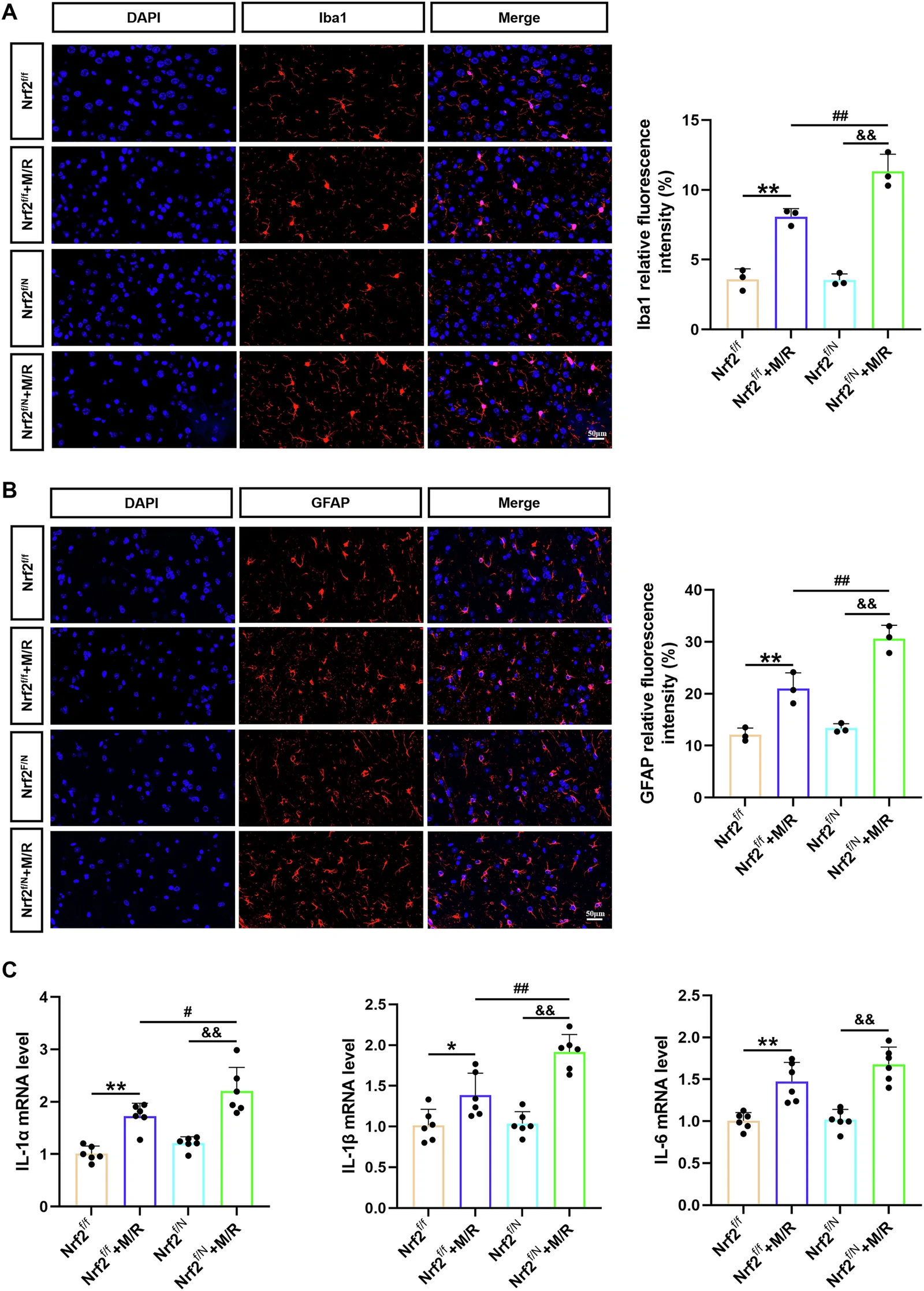
Neural lineage Nrf2 knockout promotes glial cell activation and neuroinflammation in AIS mouse models. **A** Immunofluorescence staining of Iba1 (red) and DAPI (blue) in the cerebral ischemic region. Scale bar = 50 µm (*n* = 3/group). **B** Immunofluorescence staining of GFAP (red) and DAPI (blue) in the cerebral ischemic region. Scale bar = 50 µm (*n* = 3/group). **C** Effect of neural lineage Nrf2 knockout on mRNA expression of *IL-1α*, *IL-1β*, and *IL-6* in brain tissue (*n* = 6/group). ^*^P < 0.05, ^**^P < 0.01 vs. Nrf2^f/f^ group; ^& &^*P* < 0.01 vs. Nrf2^f/N^ group; ^##^*P* < 0.01 vs. Nrf2^f/f^ + M/R group.

Glial cell-mediated neuroinflammation drives AIS pathology [27]. To investigate the role of neural lineage Nrf2 in AIS-associated neuroinflammation, the expression of glial activation markers and inflammatory cytokines was assessed. Immunofluorescence staining (Fig. 7A, B) revealed higher Iba1 and GFAP expression in the ipsilateral hemisphere of Nrf2^f/f^ + M/R mice than that of Nrf2^f/f^ controls. In the Nrf2^f/N^ + M/R group, these activation markers were further upregulated indicating that neural lineage Nrf2 deficiency amplifies AIS-driven microglial and astrocytic activation. RT-qPCR analysis revealed elevated *IL-1α*, *IL-1β*, and *IL-6* mRNA expression in the Nrf2^f/f^ + M/R group, with *IL-1α* and *IL-1β* further increased in the Nrf2^f/N^ + M/R group, suggesting a more severe neuroinflammatory response (Fig. 7C). These findings implicate the absence of Nrf2 in the neural lineage exacerbates neuroinflammation post-AIS by promoting glial activation and the release of inflammatory factors. The model’s genetic design, coupled with cell localization data, leads us to conclude that neuronal Nrf2 deficiency is the principal factor driving this aggravated response.

### Astrocyte-biased Nrf2 knockout promotes A1-type reactive astrocyte polarization in acute ischemic stroke mouse models

**Fig. 8:**
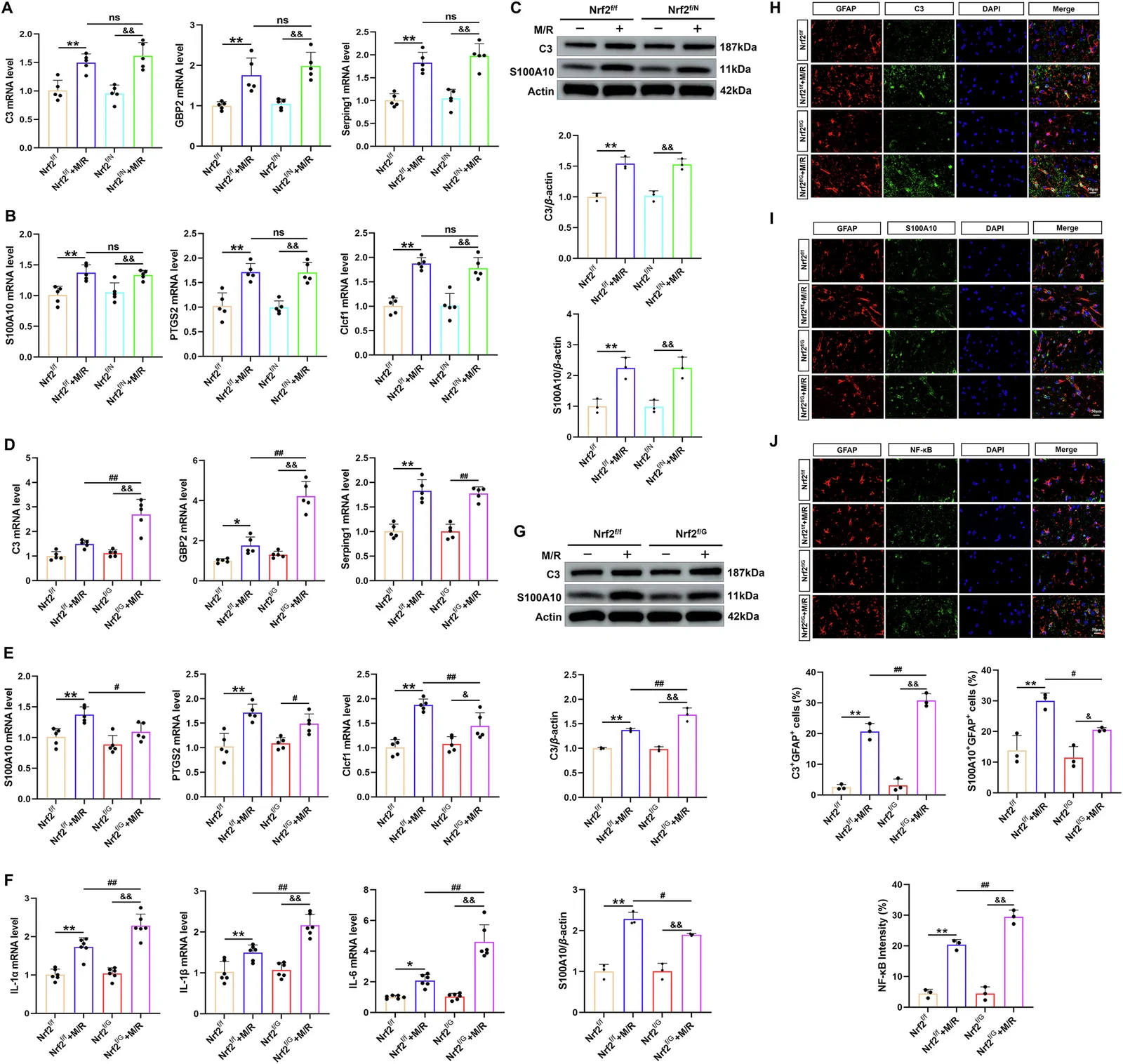
Astrocyte-biased Nrf2 knockout promotes A1-type reactive astrocyte polarization in AIS mouse models. **A** , **B** Effects of neural lineage Nrf2 knockout on A1/A2 marker expression (*n* = 5/group). **C** Western blot analysis of C3 and S100A10 protein expression after neural lineage Nrf2 knockout (*n* = 3/group). **D** , **E** Effects of astrocyte-biased Nrf2 knockout on A1/A2 marker expression (*n* = 5/group). **F** Effect of astrocyte-biased Nrf2 knockout on *IL-1α*, *IL-1β*, and *IL-6* mRNA expression (*n* = 6/group). **G** Western blot analysis of C3 and S100A10 protein expression after astrocyte-biased Nrf2 knockout (*n* = 3/group). **H** Immunofluorescence staining of astrocytes (red) and C3 (green) in the ischemic hemisphere. Scale bar = 50 μm (*n* = 3/group). **I** Immunofluorescence staining of astrocytes (red) and S100A10 (green) in the ischemic hemisphere. Scale bar = 50 μm (*n* = 3/group). **J** Immunofluorescence staining of astrocytes (red) and NF-κB (green) in the ischemic hemisphere. Scale bar = 50 μm (*n* = 3/group). ^*^P < 0.05, ^**^P < 0.01 vs. Nrf2^f/f^ group; ^&^*P* < 0.05, ^& &^*P* < 0.01 vs. Nrf2^f/N^ or Nrf2^f/G^ group; ^#^*P* < 0.05, ^##^*P* < 0.01 vs. Nrf2^f/f^ + M/R group.

Reactive astrocytes are activated after cerebral ischemia and can differentiate into neurotoxic A1 or protective A2 subtypes [28]. To assess the role of neural lineage and astrocyte-biased Nrf2 in polarization, A1 (*C3*, *GBP2*, *Serping1*) and A2 (*S100A10*, *PTGS2*, *Clcf1*) markers were measured. In the Nrf2^f/f^ + M/R group, RT-qPCR showed increased expression of A1 and A2 markers, indicating bidirectional astrocyte activation post-ischemia. Whereas the Nrf2^f/N^ + M/R group showed no significant changes in astrocyte polarization markers (Fig. 8A–C), the Nrf2^f/G^ + M/R group exhibited a pronounced shift toward the A1 neurotoxic phenotype. This shift was evidenced by the upregulation of A1 markers (*C3*, *GBP2*) and concurrent downregulation of A2 markers (*S100A10*, *Clcf1*) at the transcriptional level, along with elevated pro-inflammatory cytokines (*IL-1α*, *IL-1β*, *IL-6*) (Fig. 8D–F). Consistent with these findings, Western blot and immunofluorescence analyses confirmed increased C3 and decreased S100A10 protein expression (Fig. 8G–I). To explore the underlying regulatory mechanism, we examined the NF-κB pathway. NF-κB expression further increased in the Nrf2^f/G^ + M/R group compared to that in the Nrf2^f/f^ + M/R group (Fig. 8J). Based on this result and the observed polarization shift, we therefore propose that astrocyte-biased Nrf2 deficiency promotes A1 polarization and exacerbates neuroinflammation post-AIS, at least in part through hyperactivation of the NF-κB pathway. This mechanism appears to be specific to astrocytic Nrf2, as it was not engaged upon neural lineage Nrf2 deletion.

**Fig. 9:**
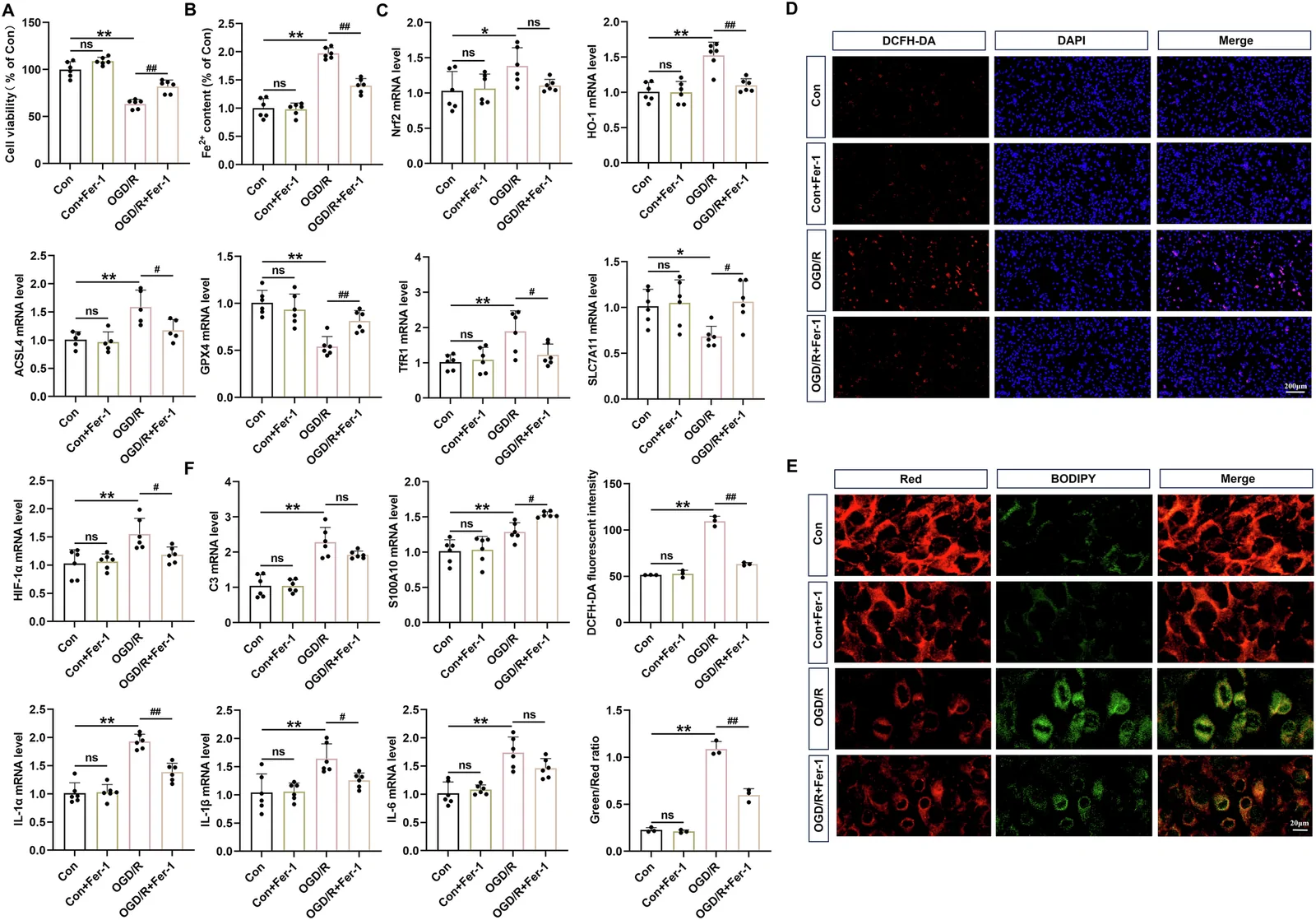
OGD/R induces neuronal ferroptosis and neuroinflammation in neurons. **A** Cell viability was assessed using the CCK-8 assay (*n* = 6/group). **B** Intracellular Fe²⁺ content (*n* = 6/group). **C** mRNA expression levels of *Nrf2*, *HO-1*, *ACSL4*, *GPX4*, *TfR1*, *SLC7A11*, and *HIF-1α* were quantified determined using RT-qPCR (*n* = 6/group). **D** Intracellular ROS levels (*n* = 3/group). **E** Lipid peroxidation levels (*n* = 3/group). **F** mRNA expression levels of *C3*, *S100A10*, *IL-1α*, *IL-1β*, and *IL-6* determined using RT-qPCR (*n* = 6/group). ^*^P < 0.05, ^**^P < 0.01 vs. Con group; ^#^*P* < 0.05, ^##^*P* < 0.01 vs. OGD/R group.

### Oxygen-glucose deprivation/reoxygenation induces neuronal ferroptosis and neuroinflammation

An in vitro co-culture of HT-22 neurons and C8-D1A astrocytes was established to validate the pathological links between AIS, ferroptosis, and neuroinflammation. The oxygen-glucose deprivation/reoxygenation (OGD/R) model was used to simulate ischemic injury in vitro, followed by Fer-1 treatment. Cell viability, ferroptosis, neuroinflammation, and oxidative stress-related markers were systematically assessed to determine the pathological effects of OGD/R and Fer-1 intervention. CCK-8 assay showed that OGD/R significantly reduced cell viability in the co-culture system compared to controls, while Fer-1 treatment increased cell viability, suggesting that inhibiting ferroptosis alleviates OGD/R-induced cell injury (Fig. 9A). RT-qPCR analysis revealed that OGD/R decreased the mRNA expression of ferroptosis-related genes *GPX4*, *SLC7A11*, while upregulating *Nrf2*, *HO-1*, *ACSL4*, *TfR1*, and *HIF-1α* expression. In the OGD/R group, intracellular Fe²⁺ levels, reactive oxygen species (ROS) generation, and lipid peroxidation also increased. Fer-1 treatment reversed these changes, confirming that OGD/R induces neuronal ferroptosis (Fig. 9B−E). Concurrently, OGD/R increased mRNA expression of neuroinflammatory factors (*IL-1α*, *IL-1β*, *IL-6*) and both A1 (*C3*) and A2 (*S100A10*) astrocyte markers, indicating astrocyte activation and inflammatory response. Fer-1 treatment reduced *IL-1α*, *IL-1β* mRNA expression, indicating that ferroptosis inhibition also mitigates neuroinflammatory response (Fig. 9F). In summary, OGD/R can induce neuronal ferroptosis and neuroinflammation, and Fer-1 alleviates both processes, suggesting that ferroptosis and neuroinflammation may act through synergistic pathological mechanisms to promote cellular injury in ischemic brain damage.

### Nrf2 knockdown differentially induces neuronal ferroptosis and astrocytic polarization, exacerbating oxygen-glucose deprivation/reoxygenation injury

**Fig. 10:**
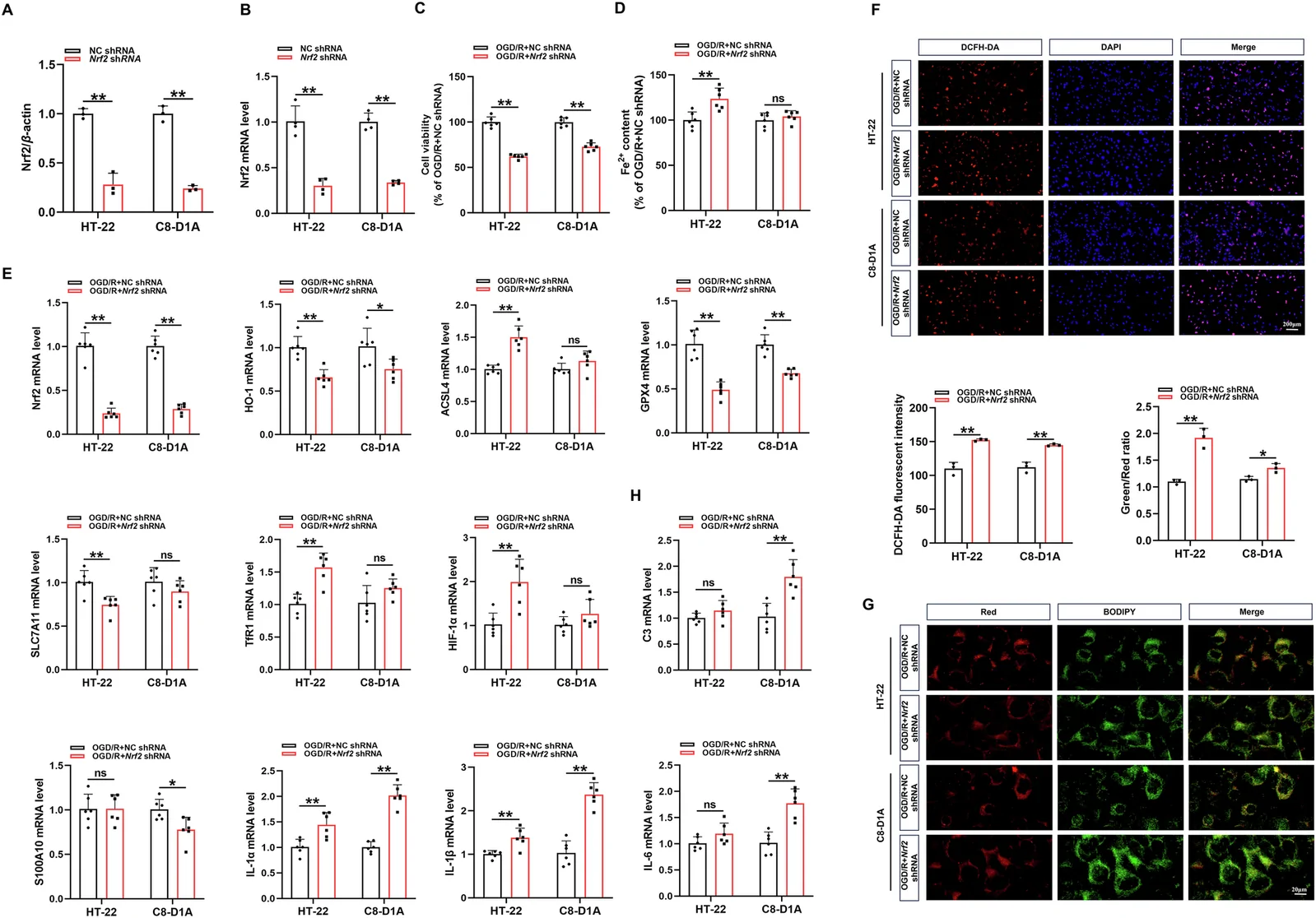
Nrf2 knockdown selectively induces neuronal ferroptosis and astrocytic polarization, exacerbating OGD/R injury. **A** Representative Western blot showing the expression of Nrf2 protein (*n* = 3/group). **B** *Nrf2* mRNA expression in HT-22 and C8-D1A cells was determined using RT-qPCR (*n* = 4/group). **C** Cell viability was evaluated using the CCK-8 assay (*n* = 6/group). **D** Intracellular Fe²⁺ content (*n* = 6/group). **E** mRNA expression of *Nrf2*, *HO-1*, *ACSL4*, *GPX4*, *SLC7A11*, *TfR1*, and *HIF-1α* determined using RT-qPCR (*n* = 6/group). **F** Intracellular ROS levels (*n* = 3/group). **G** Lipid peroxidation levels (*n* = 3/group). **H** mRNA expression levels of *C3*, *S100A10*, *IL-1α*, *IL-1β*, and *IL-6* determined using RT-qPCR (*n* = 6/group). ^*^P < 0.05, ^**^P < 0.01 vs. NC shRNA group or OGD/R + NC shRNA group.

To elucidate the cell type-specific protective roles of Nrf2 in neurons and astrocytes, lentivirus-mediated Nrf2 knockdown was performed in HT-22 neurons and C8-D1A astrocytes, followed by OGD/R modeling and functional analysis. Figure 10A, B and Figure S3 show the efficiency of Nrf2 knockdown. CCK-8 assays showed that cell viability was significantly reduced in neuronal and astrocytic Nrf2-knockdown groups, compared to that of the OGD/R + NC shRNA group, indicating a protective role for Nrf2 in both cell types (Fig. 10C). RT-qPCR results revealed that neuronal Nrf2 knockdown significantly downregulated mRNA expression of key ferroptosis factors *GPX4* and *SLC7A11*, while upregulating *ACSL4*, *TfR1*, and *HIF-1α* expression. Elevated intracellular free iron was confirmed using the Fe²⁺ assay. These molecular changes were accompanied by increased ROS generation and lipid peroxidation, consistent with ferroptosis pathology. Furthermore, upregulation of pro-inflammatory cytokines mRNA (*IL-1α*, *IL-1β*) was observed concurrently, suggesting that ferroptosis and neuroinflammation may act synergistically to exacerbate cellular damage.

In contrast to neurons, astrocytic Nrf2 knockdown downregulated only *GPX4* mRNA, with no significant changes in *SLC7A11*, *ACSL4*, or *TfR1* expression, and intracellular Fe²⁺ levels remained unchanged. These findings indicate that the canonical ferroptosis pathway was not activated. However, the reduction in GPX4 resulted in elevated ROS and lipid peroxidation, indicating GPX4-dependent oxidative stress damage (Fig. 10D−G). Concurrently, astrocytic Nrf2 knockdown upregulated the A1 polarization marker *C3*, downregulated the A2 marker *S100A10*, and significantly increased *IL-1α*, *IL-1β*, and *IL-6* mRNA expressions (Fig. 10H). This suggests that Nrf2 deficiency may amplify inflammatory responses by promoting A1 polarization, while the resulting oxidative stress may occur independently of the ferroptosis pathway.

In summary, Nrf2 performs distinct, cell-type-specific roles in neurons and astrocytes. Neuronal Nrf2 protects primarily by inhibiting ferroptosis. Astrocytic Nrf2 primarily maintains antioxidant capacity and suppresses A1-type polarization to mitigate inflammatory damage.

## Discussion

To delineate the lineage-specific protective functions of Nrf2 in AIS, we utilized complementary in vivo MCAO/R models (Nrf2^f/f^;Nestin-Cre and Nrf2^f/f^;GFAP-Cre) and in vitro OGD/R models. Our integrated analysis uncovered a stark phenotypic dichotomy: neural lineage Nrf2 knockout exacerbated injury mainly by triggering ferroptosis and cascading neuroinflammation, while its astrocyte-biased deletion acted through aggravation of oxidative stress, induction of A1-reactive astrocytes, and alteration of the neuroinflammatory niche. These results move beyond the limitations of single-cell-type analyses, establishing that Nrf2 provides concerted protection across lineages yet through distinct, lineage-dependent mechanisms, which refines the pathological network of Nrf2 in ischemia. Of paramount importance, the absence of a ferroptosis phenotype in astrocyte-biased knockouts versus its prominence in neural knockouts positions neuronal Nrf2 as the pivotal cellular guardian against ferroptosis, with astrocytic Nrf2 operating through alternative protective avenues. This insight opens new avenues for exploring cell-type-specific pathogenesis and therapeutics in AIS.

Within the central nervous system (CNS), Nrf2 exhibits distinct expression patterns across neurons, glial cells, and endothelial cells. Single-cell RNA sequencing in mice and humans reveals significantly lower Nrf2 expression in neurons compared to glial cells [29]. Moreover, Nrf2 is dynamically regulated in a cell-type-, time-, and region-specific manner across neurological disease models, and its activators exert cell-selective effects [30–32]. Defining the lineage-specific functions of Nrf2 is thus critical for dissecting disease mechanisms and advancing targeted therapeutic strategies. In IS, endogenous Nrf2 expression peaks at 24 h post-ischemia, predominantly in neurons, followed by astrocytes [33, 34], consistent with observations in the AIS mouse model. Building on the lineage-specific Nrf2 expression profile in AIS, this study further focused on neural lineage and astrocyte-biased deletion models, investigating the consequences of their respective Nrf2 deficiencies. Nrf2 deletion in either cell type exacerbated neurological deficits, increased infarct volume, and reduced CBF, and Nrf2 knockdown in vitro decreased cell viability. These findings confirm that Nrf2 exerts a critical protective effect in neurons and astrocytes.

Ferroptosis, a regulated form of cell death characterized by dysregulated iron homeostasis and accumulated lipid peroxidation, contributes to neuronal death in IS and is potentially a disease biomarker and therapeutic target [35–38]. The transcription factor Nrf2 is a master regulator of cellular antioxidant responses and protects against CI/RI by inhibiting oxidative stress, ferroptosis, and neuroinflammation, highlighting its potential as a therapeutic target for IS [39–41]. This study examined the cell-specific regulatory role of Nrf2 in ferroptosis within specific neural lineages. The results show that neural lineage Nrf2 knockout in the AIS model significantly upregulates brain Fe²⁺ content and lipid peroxidation levels. This change was accompanied by altered expression of key ferroptosis regulators, and the appearance of typical ferroptosis ultrastructural changes (e.g., mitochondrial shrinkage, increased membrane density). The ferroptosis-specific inhibitor Fer-1 reversed these pathological alterations, confirming that neural lineage Nrf2 deficiency exacerbates injury via ferroptosis activation. In contrast, astrocyte-biased Nrf2 knockout exacerbated oxidative stress damage but did not induce ferroptosis-associated iron metabolism dysregulation and lipid peroxidation burst. In vitro OGD/R experiments further confirmed this lineage-dependent phenomenon. These findings elucidate a lineage-dependent protective mechanism of Nrf2 in AIS: genetic ablation-based comparative analyses indicate that neuronal Nrf2 likely serves as a key contributor to ferroptosis suppression, whereas astrocytic Nrf2 primarily exerts its protective effects through augmentation of antioxidant defenses. Ischemic brain injury involves the synergistic activation of multiple cell death pathways, including apoptosis, necroptosis, autophagy, and pyroptosis [42]. Existing evidence suggests that in early-stage acute ischemia-reperfusion, ferroptosis—driven by iron overload and lipid peroxidation—may initiate a cascade involving other cell death pathways [41, 43, 44]. For instance, oxidative stress from ferroptosis can trigger necroptosis, while excessive autophagy may induce ferroptosis by inducing iron overload [45, 46]. Therefore, neuronal damage observed in this study may be triggered early by ferroptosis and subsequently intertwining with other cell death mechanisms to collectively exacerbate the brain injury process. While future studies using inhibitors that target multiple cell death pathways are warranted for more precise dissection, the rescue experiments presented here suggest that targeting the neuronal Nrf2–ferroptosis axis may offer novel, lineage-biased neuroprotective strategies.

HIF-1α is a master regulator of the cellular hypoxic response. Moderate HIF-1α activation under acute hypoxia promotes adaptive cellular responses, whereas sustained high levels induce cellular damage [47]. The expression and function of HIF-1α exhibit significant lineage specificity across disease contexts and can influence disease progression by modulating ferroptosis [48–50]. Our results indicate that Nrf2 deficiency in the two genetically defined models exerted differential impacts on HIF-1α expression in AIS model mice. Neural lineage Nrf2 knockout significantly increased HIF-1α expression, whereas astrocyte-biased Nrf2 knockout had no significant effect. Mechanistic studies suggest that HIF-1α is a key regulator of TfR1. HIF-1α upregulation can induce TfR1 overexpression, enhance extracellular iron influx, and promote Fe²⁺ accumulation, thereby triggering ferroptosis [51–54]. TfR1 expression and Fe²⁺ levels were upregulated in the AIS model mice. These increases were further enhanced following neural lineage Nrf2 knockout but remained unchanged with astrocyte-biased Nrf2 knockout. These findings suggest that neuronal Nrf2 may influence Fe²⁺ homeostasis and subsequently contribute to ferroptosis activation by regulating the HIF-1α/TfR1 signaling pathway. Astrocytic Nrf2, however, likely functions independently of this pathway. This lineage-specific regulatory pattern suggests a molecular basis for differential Nrf2-mediated regulation of ferroptosis in AIS.

Neuroinflammation is another critical pathological mechanism underlying neural injury in IS. Astrocyte- and microglia-mediated neuroinflammation operates across all stages of IS, from early pro-inflammatory induction of tissue damage to later anti-inflammatory processes involved in tissue repair [55]. Evidence suggests a bidirectional relationship between ferroptosis and neuroinflammation. Inflammatory responses impair antioxidant defenses and promote iron and lipid peroxide accumulation, thereby facilitating inflammation-induced ferroptosis. Conversely, lipid peroxides and damage-associated molecular patterns released during ferroptosis amplify neuroinflammation [56, 57]. In this study, neural lineage Nrf2 knockout potentiated glial activation and pro-inflammatory mediator release in AIS mice. It is noteworthy that, given the theoretical framework of Nestin-Cre-mediated recombination, the observed exacerbation of glial activation may reflect a confluence of the primary effect of neuronal Nrf2 deletion and potential secondary effects arising from Nrf2 deficiency in glial cells. However, it remains unresolved whether neuronal Nrf2 deletion enhances neuroinflammation that secondarily induces ferroptosis, accelerates ferroptosis that in turn amplifies neuroinflammation, or simultaneously engages in a bidirectional feedforward loop. Therefore, clarifying the temporal and causal relationships among these processes, beyond the limitations of the Nestin-Cre model, warrants further investigation using more precise, lineage-restricted tools.

Evaluation of Nrf2 deficiency across distinct genetically targeted models revealed a cell-type-specific impact on astrocyte reactivity and neuroinflammation. While neural lineage Nrf2 knockout (in vivo AIS model) and neuronal-specific Nrf2 knockdown (in vitro OGD/R model) solely increased the release of pro-inflammatory mediators without significantly altering the gene or protein expression of the reactive astrocyte markers C3 and S100A10. In contrast, astrocyte-biased Nrf2 knockout significantly upregulated the A1-type astrocyte marker C3 and downregulated the A2-type marker S100A10 at mRNA and protein levels in AIS mouse brains. These findings indicate that astrocytic Nrf2 deficiency promotes a pro-inflammatory A1 astrocyte phenotype, whereas neural lineage Nrf2 deficiency likely exacerbates neuroinflammation via pathways independent of direct astrocyte polarization. NF-κB, a master transcription factor regulating immune and neuroinflammatory responses, promotes A1 astrocyte activation by driving the transcription of inflammatory genes [58]. Furthermore, extensive molecular crosstalk between Nrf2 and NF-κB regulates glial cell polarization under stress and inflammatory conditions [59]. Consistent with previous studies [60], astrocyte-biased Nrf2 knockout enhanced NF-κB expression. These observations suggest that astrocytic Nrf2 mitigates neuroinflammatory damage by suppressing the NF-κB signaling pathway and inhibiting A1 astrocyte activation. Conversely, neural lineage Nrf2 likely mediates anti-inflammatory effects via mechanisms independent of the NF-κB/astrocyte axis. This lineage-specific regulatory pattern further elucidates the mechanisms by which Nrf2 differentially mediates neuroinflammation in AIS at the molecular level.

The core contribution of this study is its systematic comparison of the differential protective roles of neural lineage and astrocyte-biased Nrf2 signaling in AIS. For model generation, the widely utilized Nestin-Cre and GFAP-Cre driver lines were employed to achieve neural lineage and astrocyte-biased Nrf2 knockout, respectively. A critical consideration is that Nestin-Cre acts on neural precursors, affecting multiple neural lineages. The immunofluorescent mosaic of Nrf2 expression in astrocytes confirms this complexity. We therefore define our model as a broad neural-lineage Nrf2 deletion and rationally inferred neuron-specific functions by contrasting phenotypes with the GFAP-Cre model—a subtractive comparative approach. This paradigm leverages the tool’s actual properties for valid inter-lineage comparison. Subsequent work using more specific tools (e.g., Camk2a-Cre, Synapsin I-Cre) is needed to isolate specific cellular contributions and directly test the conclusions derived from our model-based comparison. Mechanistically, although differential regulation of HIF-1α and TfR1 by the two genetically defined models was observed, whether this interaction directly mediates their heterogeneous effect on ferroptosis requires further verification using pathway-specific interventions. While neural lineage Nrf2 deficiency did not significantly alter reactive astrocyte polarization status, its potential indirect role in neuroinflammation via modulating microglial polarization (e.g., M1/M2 balance) remains unclear, and whether astrocytic Nrf2 reciprocally regulates microglial function remains a compelling scientific question worthy of further exploration. Additionally, the roles of Nrf2 in oligodendrocytes and endothelial cells, along with its spatiotemporal signaling interaction network of Nrf2 across different neural lineages, constitute important directions for future research. Addressing these questions will facilitate the development of a comprehensive, lineage-dependent Nrf2 regulatory network, thereby providing a stronger theoretical foundation for precisely targeted therapeutic strategies.

## Conclusion

In conclusion, this study demonstrates that Nrf2 exerts lineage-dependent protection in AIS. Through comparative analysis of neural lineage and astrocyte-biased knockout models, we found that while Nrf2 deficiency in both genetically defined models exacerbate brain injury, they exhibit significant phenotypic divergence. Neural lineage Nrf2 deficiency primarily exacerbates injury by promoting ferroptosis and neuroinflammation. In contrast, astrocyte-biased Nrf2 deficiency mainly enhances oxidative damage, shifts astrocytes toward an A1-polarized phenotype, and amplifies neuroinflammation. Utilizing a subtractive comparative logic, we rationally infer that neuronal Nrf2 serves as a key contributor to inhibiting ferroptosis in acute ischemic brain injury, while astrocytic Nrf2 predominantly modulates the neuroinflammatory microenvironment via the NF-κB pathway. These findings elucidate the molecular basis of functional heterogeneity of Nrf2 across distinct neural lineages and provide a critical theoretical foundation and novel perspectives for developing lineage-biased Nrf2-targeted intervention strategies.

## Supplementary information

Supplementary Information

## Data Availability

The data supporting the findings of this study are available within the article and its Supplementary Materials. Additional raw datasets can be obtained from the corresponding author upon reasonable request.
