# Supplementary material for: Lineage-specific Nrf2 signaling orchestrates distinct neuroprotective mechanisms in acute ischemic stroke
Source: Cell Death Dis. 2026 Jul 13;17(1):870. doi: 10.1038/s41419-026-09047-8 (PMC13645789; doi:10.1038/s41419-026-09047-8)
Supplement: Supplementary file 1 [file 41419_2026_9047_MOESM1_ESM.docx]

**Supplementary figure legends**

**Figure S1** **Validation of the Nrf2 gene knockout in neurons and astrocytes.**

(A) Assessment of Nrf2 expression via immunofluorescence in neurons and astrocytes of Nrf2^flox/flox^; Nestin-Cre (Nrf2^f/N^) mice following AIS. White and red arrowheads indicate Nrf2-positive astrocytes and neurons, respectively (Scale bar = 20 µm, n = 3/group).

(B) Immunofluorescence analysis of Nrf2 expression in neurons and astrocytes of AIS model mice (scale bar = 50 µm, n = 3/group).

(C) Immunofluorescence staining sections of the cerebral cortex of Nrf2^f/f^ and Nrf2^f/N^ mice, showing neurons (red) and Nrf2 (green). Scale bar=50 µm, n=3/group.

(D) Immunofluorescence staining sections of the cerebral cortex of Nrf2^f/f^ and Nrf2^f/G^ mice, showing astrocytes (red) and Nrf2 (green). Scale bar=50 µm, n=3/group.

(E) RT-qPCR analysis of *Nrf2* mRNA expression in the cerebral tissues of Nrf2^f/f^ and Nrf2^f/N^ mice(n=6/group).

(F) RT-qPCR analysis of *Nrf2* mRNA levels in the cerebral tissues of Nrf2^f/f^ and Nrf2^f/G^ mice (n=6/group).

^**^*P*<0.01 vs. Nrf2^f/f^ group.

**Figure S2.** **Effects of astrocyte-biased Nrf2 knockout on Fe²⁺ and lipid peroxidation in the AIS mouse model.**

(A) Fe²⁺ content in the cerebral ischemic region (n=5/group).

(B) SOD activity (n=6/group).

(C) MDA levels (n=5/group).

(D) GSH/GSSG ratio (n=5/group).

^**^*P* < 0.01 vs. Nrf2^f/f^ group; ^&&^*P* < 0.01 vs. Nrf2^f/G^ group; ^#^*P* < 0.05 vs. Nrf2^f/f^ + M/R group.

**Figure S3.** Western blot analysis of Nrf2 protein expression levels in HT-22 and C8-D1A cells (n=3/group).

**Figure S1**

**Figure S2**

**Figure S3**

**Uncropped gels for Western blot in Figure 4A**

**Nrf2** **NQO1**

 **HO-1**

 **ACSL4**

 **4-HNE**

 **GPX4**

 **TfR1**

**Uncropped gels for Western blot in Figure 4B**

 **Nrf2**

 **NQO1**

 **HO-1**

 **ACSL4**

 **4-HNE**

 **GPX4**

 **TfR1**

**Uncropped gels for Western blot in Figure 6C**

 **HIF-1α**

**Uncropped gels for Western blot in Figure 6D**

 **HIF-1α**

**Uncropped gels for Western blot in Figure 8C**

 **C3**

 **S100A10**

**Uncropped gels for Western blot in Figure 8G**

 **C3**

 **S100A10**

**Uncropped gels for Western blot in Figure S3**

 **Nrf2(HT-22)**

 **Nrf2(C8-D1A)**
